# Psychological predictors of the time perspective: The role of posttraumatic stress disorder, posttraumatic growth, and temporal triggers in a sample of bereaved adults

**DOI:** 10.1371/journal.pone.0298445

**Published:** 2024-03-01

**Authors:** Leia Y. Saltzman, Lauren Terzis

**Affiliations:** 1 School of Social Work, Tulane University, New Orleans, LA, United States of America; 2 School of Health Sciences and Social Work, Griffith University, Brisbane, Australia; Universitat d’Alacante, SPAIN

## Abstract

**Introduction:**

The process of coping with loss and trauma is inextricably linked with subjective experiences and perceptions of time. The Time Perspective Framework, suggests that temporal frames influence an individual’s actions, judgements, and the decisions that they make. Similarly, time perspective has been linked with psychosocial outcomes of trauma and loss (e.g. posttraumatic stress disorder). The aim of this study is to identify factors that may influence survivor’s time perspective in order to determine if posttraumatic stress disorder (PTSD), posttraumatic growth (PTG), and temporal triggers (MIT) influenced different time perspective orientations.

**Methods:**

Data was collected via Qualtrics survey between July 2019 and July 2020. Measures included basic demographic characteristics, posttraumatic stress disorder checklist, posttraumatic growth inventory, an assessment of temporal triggers, and the Zimbardo time perspective inventory. A series of OLS regression analyses were estimated.

**Results:**

PTG was positively associated with future time perspective scores, whereas PTSD was associated with an increase in past negative orientation. The endorsement of temporal triggers like markers in time was negatively associated with present hedonistic scores and positively associated with future time perspective scores. Interestingly, PTSD, PTG and the endorsement of MIT were not associated with present time perspective scores.

**Discussion and implications:**

Identifying the relationship between PTSD, PTG, marker in time, and time perspective may offer important insights into treatment modalities that can improve outcomes for bereaved individuals. We discuss limitations of the current analysis and make recommendations for future research.

## Introduction

The loss of a loved one is a universal experience, but how an individual copes with that loss will depend on many factors [1]. The nature of the loss influences the process of coping afterwards. For example, deaths that are traumatic–that is “off time” sudden or violent—may initiate a more tumultuous process of coping than an expected loss in which loved ones had time to prepare [2, 3]. Similarly, the relationship held with the deceased loved one can also influence the experience of grief [4] such that close relationships are more likely to trigger more negative psychological and emotional reactions [5]. While many individuals will resolve the experience of grief and bereavement by relying on their existing coping skills and natural supports; others will struggle with the psychological, physical, behavioral, and social reactions to loss [1, 6].

### Outcomes of loss and bereavement

#### Posttraumatic stress disorder

Posttraumatic stress disorder (PTSD) is most commonly linked with trauma exposure. However, the Diagnostic and Statistical Manual (DSM) includes the loss of life or threatened loss of life as a component of criteria A—which outlines the context for traumatic exposure [7]. PTSD as a disorder, includes four symptom clusters: (1) re-experiencing; (2) avoidance; (3) negative cognitions and emotions; and (4) hyperarousal that result in functional impairment at least six months following the event [7]. Violent death [8], sudden unexpected death [9–11], and a loss by suicide [12] have been associate with PTSD. PTSD, in turn, is comorbidly associated with other negative mental health outcomes of loss such as depression, anxiety, and substance use disorders [13]. While any loss of a loved one has the possibility of being internalized as a traumatic event; sudden or violent deaths pose a unique context in which to understand occurrences of PTSD. More specifically, context of traumatic loss includes both the typical experience of bereavement, but also incorporates a trauma reaction requiring the survivor to not only cope with grief but also with processing the traumatic exposure associated with it [2]. Factors such as identifying as female, pre-existing mental health challenges, nature of the loss, and social isolation have been noted to increase the risk of PTSD following traumatic loss [3].

#### Posttraumatic growth

While some individuals will experience negative mental health outcomes such as PTSD, others report positive adaptation trajectories such as resilience and even growth [14]. Posttraumatic growth (PTG) refers to the positive adaptations that can be reported by survivors as a result of the process of coping with trauma and/or loss [15, 16]. PTG is typically expected in five areas of life including: (1) appreciation of others; (2) greater sense of personal strength; (3) closer relationships; (4) perception of new opportunities; and (5) spirituality or religion [15]. In recent years, PTG has become a controversial indicator of wellbeing following trauma; with some scholars noting the complex relationship between PTG and other indicators of trauma adaption [17–20]. More specifically, conflicting reports regarding the relationship between PTG and PTSD have been documented with some finding and inverse relationship [21], others a curvilinear relationship [22, 23], and still others no relationship at all [24].

In our own work, we follow the conceptualization of the Janus Face Model proposed by Maercker and Zoellner [24, 25] purporting two components of PTG. The first, illusory growth is thought to be compensatory, that is finding “benefit” is psychologically advantageous to the survivor and allows them to better cope with the trauma or loss. Alternatively, constructive growth, is thought to develop over time and be related to other indicators of wellbeing. In our work we note that PTSD and PTG are not mutually exclusive and have found that both can be reported simultaneously in a profile we refer to as “struggling growth” [17, 18]. As in the trauma literature, PTG has been documented in experience of bereavement including traumatic loss [26, 27]. Factors such as time since the loss, coping skills, social support, religiosity, relationship with deceased, and age are predictive of PTG following loss [26, 27].

### Time and bereavement

Grief that extends for prolonged periods of time has been referred to the literature by several names; complicated grief, prolonged grief, and complex bereavement [28]. Complicated grief occurs when the bereaved individual is unable to effectively process and integrate the loss of a loved one, extending their experience of mourning and grief for a prolonged time; by diagnostic standards more than 12 months (6 months for a child) following the loss [7, 29, 30]. Complicated grief can lead to distressing and intrusive thoughts after the death, as well as disbelief and negative feelings and emotions [30]. Complicated grief impacts the bereaved individual’s ability to function in their daily life, without the constant reminder and associations to the loss [30].

Regardless of the label, all of these “disorders” assume that grief should resolve within a specific timeframe and failing to do so should be considered a pathological response falling outside of the typical reaction to loss, warranting a diagnostic label. To some extent, this approach is rooted in traditional frameworks of grief and bereavement. For decades, the prevailing approach to understanding loss was based on the seminal work of Kubler-Ross’s five stage model of grief. These stages include: 1) denial, 2) anger, 3) bargaining, 4) depression, and 4) acceptance [31]. There has been much criticism and controversy surrounding Kubler-Ross’s stages. The term “stage” has been debated, as it implies that the individual follows a linear step process, when the lived experience of loss is often more complex. This has led to a softening of stage models, and a greater focus on the cyclical approaches to bereavement [32, 33].

While cyclical models offer a more accurate representation of the lived experience of grief, they lack emphasis on the role that *subjective* time plays in bereavement and loss. To address this gap, our previous work [33, 34] posits a reconceptualized framework that shifts the narrative to look at *meaningful time*. The meaningful time approach moves away from the traditional linear progression of absolute time, where time is even and chronological, and instead, focusses on a more cyclical and subjective approach to understanding the role of time. One in which, time is uneven, and interacts with the individual’s personal, cultural and historical contexts [33].

### Time informed approaches to bereavement and trauma

The meaningful time approach includes the notion of *markers in time (MIT)*, which is an expansion to traditional definitions of anniversary reactions. These markers can include important events linked to the traumatic loss, and are experienced as inevitable, repeated temporal triggers that are significant because they are meaningful to the survivor [33]. Markers are also deeply emotional and often associated with bereavement rituals (e.g. visiting the grave, attending a religious service, or gathering with friends and family) [33, 35]. Markers may include cultural and social events that occur each year, such as birthdays, holidays, as well as other moments in time that are uniquely linked with the death experience. There are three discrete phases including, an anticipatory phase, the marker itself, and a recovery phase; mimicking an arch like pattern [33, 35]. Markers in time are significant in that they disrupt the individual’s perception of time, and the bereaved must reorient their process of coping around these temporal triggers [33]. Over time, it is thought that the phases surrounding the marker may become more condensed and abbreviated (e.g. over time recovery happens faster after a marker).

Like the meaningful time approach, Zimbardo’s time perspective (TP) recognizes that time has a great influence on the individual experience and human functioning. In his theory, Zimbardo, split time into *temporal frames* including past, present, and future [33, 36]. These temporal frames are further divided into six categories: past negative, past positive, present hedonism, present fatalism, future, and transcendental future [36, 37]. Zimbardo and Boyd suggest that the time perspective and temporal frames influence an individual’s actions, judgements, and the decisions that they make [36]. For example, recalling and focusing on the past can impact one’s response to a current situation, as well as expectations and anticipation for the future [36]. Ideally, an individual would have a “balanced time perspective”, meaning that they are able to navigate between the three temporal frames (past, present, and future) without issue [36].

Research has found that the time perspective can be helpful when applied to trauma, specifically with individuals that are diagnosed with PTSD. Having a balanced time perspective has found to lead to higher levels of happiness and wellbeing [38], and mediates the relationship between trauma exposure, temperament, and PTSD symptoms [39, 40]. On the other hand, if the individual does not have a balanced time perspective, this can lead to rumination in the past, lower levels of optimism and distortion in thinking [39]. Specifically, individuals that had a negative time perspective bias have been found to have a harder time coping with their trauma, thus leading to more intense PTSD symptoms [40].

This approach may also have important clinical application. Zimbardo developed Time Perspective Therapy (TPT) as way in which to treat clients diagnosed with PTSD rooted in the temporal frames proposed by the Time Perspective framework. The time perspective can also be useful in understanding post-traumatic growth (PTG) among those that have experienced a horrific event, such as traumatic loss. One’s focus on future time orientation may lead them to be more likely to develop post-traumatic growth after a loss [41]. Individuals that have a future time orientation have been found to be more motivated to enact positive change and new possibilities, and engage in meaning-making, all elements of posttraumatic growth [41].However, studies regarding the relationship between the time perspective and PTG remain few in number. Given the complex relationship between PTSD and PTG, and the documented relationship between PTSD and the time perspective, we argue that further exploring the relationship between PTG and the time perspective is warranted. Furthermore, we suggest that understanding how scores on more established measures like PTSD and PTG may influence time perspective orientation may have important clinical implications for practice with trauma affected individuals, families, and communities.

### Current study

The influence of temporal elements in the process of coping with loss is gaining traction in the scholarly literature and has vast implications for the treatment of bereaved individuals struggling to cope with experiences of loss. The scope of the project was to explore the perceptions of time and identify the unique experiences of adults coping with bereavement. The current analysis uses a subsample of a larger data file to explore the influences of posttraumatic stress, posttraumatic growth, and markers in time on the time perspective. We describe the selection of this sub-sample in greater detail below. The aim of this sub-sample analysis is to identify factors that may influence survivor’s time perspective in order to determine if a relationship between these constructs exists. Identifying the relationship between PTSD, PTG, marker in time, and time perspective may offer important insights into treatment modalities that can improve outcomes for bereaved individuals.

## Methods

### Procedures

Data was collected via Qualtrics survey between July 2019 and July 2020. A flyer describing the study was posted and/or shared in various forums including social media platforms. Participants who were interested could click on an embedded link that directed them to the Qualtrics survey. Participants were 18 years of age or older, comfortable completing a survey in English, and could not have experienced the loss of loved one within six months of enrollment. Upon completion of the survey participants received a $10 electronic gift certificate. In the Qualtrics form, participants viewed a welcome letter describing the study aims and tasks. Once completed participants were routed to a consent script.

### Ethics statement

The consent script was approved by the University IRB. Participants were able to review the consent script and had two options at the bottom of the page, one indicating consent to participation and the second declining to consent. If participants clicked on the “consent” option they were routed to the study, if they declined to consent, they were routed to a “thank you” page that contained the investigators contact information. Study procedures were approved by Tulane University Human Research Protection Office study #2018–2132.

### Measures

Participants completed basic demographic information including: (1) age, (2) gender; (4) racial/ethnic background; (5) socioeconomic status; and (6) marital status.

### Time perspective

The Zimbardo Time Perspective Inventory (ZTPI) [36] was used to assess time perspective. The ZTPI includes 15 items that ask participants to rate the degree to which the statement is true about them on a 5-point Likert scale with responses ranging from 0 “very untrue” to 4 “very true” (total α = 0.63). The scale is divided into 5 subscales based on “temporal frames” propose by Zimbardo and Boyd (1999). The temporal frames are organized into three orientations past, present and future. Past and present each have two sub-scales: one reflecting a positive time perspective and the other a negative time perspective. The future temporal frame only includes one subscale. All sub-scales are comprised of three items from the ZTPI.

We follow the same structure in our coding of the ZTPI such that the past temporal frame is comprised of two subscales past negative ("I think about the bad things from the past" "Painful experiences replayed in my” "it is hard for me to forget unpleasant image of my youth"; α = 0.71) and past positive ("familiar childhood sights, sounds, smells bring flood of wonderful memories" "happy memories of good time spring to mind" "I enjoy stories about how things used to be"; α = 0.60). The present temporal frame is comprised of two subscales present fatalistic ("life today is too complicated" "since whatever will be will be it doesn’t really matter what I do" "often luck pays off better than hard work"; α = 0.45) and present hedonistic ("I make decisions on the spur of the moment" "taking risks keeps my life from becoming boring" "it is important to put excitement in my life"; α = 0.47). While the future temporal frame, including only one sub-scale future ("when I want to achieve something I set goals and consider specific means of reaching them" "meeting tomorrows deadlines and doing other necessary work comes before tonight’s play" "I complete projects on time by making steady progress"; α = 0.72).

### Posttraumatic stress disorder

Posttraumatic Stress Disorder Checklist standard format (PCL 5-S) [42]; is a 17-item scale (total α = .91) to assess post-traumatic stress symptomology. Responses are ranked on a 5-point Likert scale with responses ranging from 0 “not at all” to 4 “extremely.” Other uses of the PCL 5 included 3 symptoms clusters: (1) re-experiencing; (2) hyperarousal; and (3) avoidance based on symptom endorsement. Given the small sample size of the current study we use the total score for our analysis to reflect the wider range of distress symptoms experienced by our sample.

### Posttraumatic growth

The Posttraumatic Growth Inventory [15] includes 10 items on a 5-point Likert scale with responses ranging from 0 “not at all” to 4” a very great degree” (total α = 0.80). The PTGI includes 5 subscales reflection arenas in which growth is expected to emerge, these include: (1) new appreciation of life; (2) closer relationships; (3) spirituality; (4) new opportunities; and (5) personal strength. Given the small sample size in our analysis we use the total score to reflect a wider range of experiences endorsed by our participants.

### Markers in time (MIT)

Two open ended questions regarding “are there certain times of the year when you miss your loved ones more than usual” and “are those times/days the always the same”. If participants responded “yes” to both questions they were coded as having endorsed the experience of markers in time [33, 35]

### Data cleaning and careless responses

Survey responses were screened for duplications. Duplicates were deleted when repeated email addresses were present (n = 15) or when qualitative responses clearly indicated copy and paste (n = 11). Unique responses were included in the final sample for the data analysis (n = 287). Responses missing 75% of the data (n = 147) were excluded, dropping the final sample size to n = 140. Of the 140, a subsample was identified using a single screening question “have you lost a close loved one within the last 10 years (closed loved one defined as parent, sibling, child, or spouse)”; 34 participants responded “yes” and these individuals comprise the subsample used for this analysis. Regression models utilized list-wise deletion.

## Results

Demographic information is reported on the full sample (n = 140) in Table 1. The sample was predominately female (n = 104; 74.29%) and white (n = 90;65.69%) with the majority of the sample endorsing average income levels (as compared to others their age) (n = 72; 51.43%) and a relationship status of either single, divorced, or widowed (n = 103; 73.57%). Of the total sample, 34 (28.33%) reported experiencing a loss within the last 10 years, 19 (57.58%) reported times of the year when they missed their loved ones more than usual, and 13 (72.22%) reported that those times of the year were always the same. We report the demographics of the 34 respondents who indicated they had experienced the loss of loved one in Table 2.

**Table 1:** Demographics full study sample.

Variable	Responses	F(%)
Sex	Male	36(25.71)
	Female	104(74.29)
	White	90(65.69)
	African American/ Black	15(10.95)
Race	Asian	13(9.49)
	Hispanic	10(7.30)
	Other Race	9(6.57)
	Lower than average	27(19.29)
Income	Average	72(51.43)
	Higher than average	41(29.29)
Relationship	Single, divorced, widowed	103(73.57)
	Married, living with someone	37(26.43)
Lost a close loved one	Yes	34(28.33)

Total Sample N = 140

**Table 2:** Demographics sub-sample with loss.

Variable	Responses	F(%)
Sex	Male	7(20.59)
	Female	27(79.41)
	White	18(52.94)
	African American/ Black	6(17.65)
Race	Asian	6(17.65)
	Hispanic	3(8.82)
	Other Race	1(2.94)
	Lower than average	6(17.65)
Income	Average	20(58.82)
	Higher than average	8(23.53)
Relationship	Single, divorced, widowed	24(70.59)
	Married, living w/ someone	10(29.41)
Temporal triggers	Yes	19(55.88)
Temporal triggers at same time of year (MIT)	Yes	13(38.24)
	Mean(SD) for continuous measure	
PTSD symptoms total score	29.06(13.47)	
PTG total score	19.94(7.32)	

Total Sample N = 34

A correlation matrix was generated for the loss subsample (n = 34) using all continuous variables this included the total score of PTSD, total score for PTG, and each of the subscales of the ZTPI including: (1) present fatalistic; (2) present hedonistic; (3) future; (4) past positive; and (5) past negative is presented in Table 3. All subscales of the ZTPI were significantly correlated with each other with the exception of the past negative and past positive subscale which would have been expected to be inversely correlated with each other. Past negative and past positive were both significantly correlated with total PTSD score, such that as PTSD scores increased past scores on past positive decreased (r = -0.53, p<0.01) and as PTSD scores increased scores on the past negative subscale increased (r = 0.42, p<0.05). Total score of PTG was only significantly correlated with the future subscale of the ZTPI such that increasing scores on the PTGI were associate with increasing scores on the future orientation subscale of the ZTPI (r = 0.41, p<0.05).

**Table 3:** Correlation matrix continuous variables.

	Present Fatalistic	Present Hedonistic	Future	Past Positive	Past Negative	PTSD	PTG
Present Fatalistic	-						
Present Hedonistic	0.40***	-					
Future	0.23**	.038***	-				
Past Positive	0.31***	0.62***	0.56***	-			
Past Negative	0.40***	.20*	0.32***	0.16	-		
PTSD	0.22	-0.06	-0.22	-0.53**	0.42*	-	
PTG	0.23	-0.11	0.41*	0.17	0.02	-0.07	-

* p<0.05

** p<0.01

***p<0.001

Once significant associations were found in the correlation matrix, separate regression models were estimated using OLS for each of the ZTPI subscales using total PTSD, total PTG, and the presence or absence of identified markers in time as predictors, results are presented in Tables 4–8 respectively. As with the correlation matrix, these regression models were estimated using the loss subsample (n = 34). The pattern of results from the correlation matrix was replicated in the regression models for total PTSD and total PTG, with total PTSD score predicting a decrease in scores on the past positive subscale (b = -0.11, p < 0.01) and an increase in scores on the past negative subscales (b = 0.10, p < 0.05). Similarly, total PTG scores were associated with a significant increase in scores on the future subscale (b = 0.11, p < 0.05). Interestingly, the endorsement of markers in time was significantly associated with decreases in total scores on the past hedonist subscale (b = -1.69, p < 0.05) and increase in total score on the future subscale of the ZTPI (b = 1.91, p < 0.05). Markers in time, PTSD, and PTG were not significant predictors of scores on the past fatalist subscale.

**Table 4:** Regression present fatalistic.

	b(SE)
PTSD total	0.03(0.04)
PTG total	0.10(0.07)
Marker in time	-1.19(1.03)

N = 31

* p<0.05

**p<0.01

**Table 5:** Regression present hedonistic.

	b(SE)
PTSD total	-0.03(0.02)
PTG total	-0.01(0.04)
Marker in time	-1.69(0.67)*

N = 31

* p<0.05

**p<0.01

**Table 6:** Regression future.

	b(SE)
PTSD total	-0.01(0.03)
PTG total	0.11(0.05)*
Marker in time	1.91(0.79)*

N = 31

* p<0.05

**p<0.01

**Table 7:** Regression past positive.

	b(SE)
PTSD total	-0.11(0.03)**
PTG total	0.05(0.06)
Marker in time	-0.82(0.87)

N = 31

* p<0.05

**p<0.01

**Table 8:** Regression past negative.

	b(SE)
PTSD total	0.10(0.04)*
PTG total	0.01(0.07)
Marker in time	1.11(1.07)

N = 31

* p<0.05

**p<0.01

## Discussion

Overall, the results of our analysis are consistent with previous literature regarding the expected relationships between PTSD, PTG, and time perspective. More specifically, PTG was positively associated with future time perspective scores, whereas PTSD was associated with an increase in past negative orientation. Interestingly, PTSD functioned differently for past positive orientation, such that PTSD was negatively associated with past positive scores. This finding suggests that while PTSD still impacts the past temporal frame it does seem to function differently for positive and negative orientations. Our analysis is the first to explore the relationship between temporal triggers like markers in time with time perspective. Our results indicate that endorsing markers in time was negatively associated with present hedonistic scores and positively associated with future time perspective scores. Interestingly, PTSD, PTG and the endorsement of MIT were not associated with present time perspective scores.

While the pool of literature regarding the time perspective is small a growing awareness regarding the relationship between trauma adaptation/ bereavement and perception of time is growing. While the majority of these studies considers time perspective to be *predictive* of mental health outcomes; our study takes an alternative approach. Instead, we focus on the ways in which exposure to loss and trauma may alter or influence our perception of time; our findings offers preliminary support for this approach.

Anecdotally, our results seem logical–that is PTSD would be associated with more negative time orientations (e.g. past negative) and may demonstrate an inverse relationship to more positive time perspectives (e.g. past positive). Alternatively, PTG, an indicator of wellbeing as a result of coping with trauma is logically connected to future, or more hopeful orientations. Beyond anecdotal logic, our findings are consistent with the limited empirical evidence that is available. To date, scholars have found that one’s time perspective is associated with PTSD, growth, and depression [43–45]. This relationship is well established to the point where a therapeutic approach that effectively addresses PTSD, anxiety and depression has been developed [37, 46, 47]. Previous studies have even found similar patterns among the subscales of the time perspective inventory. For example, in their study on survivors of car accidents, Mairean and Diaconu-Gherasim confirmed the association of PTSD with past negative time perspective and suggested locus of control as a possible mediator for this relationship [45]. Similarly, in a sample of emerging adults, Arpawong and colleagues found PTG was associated with future time perspectives [41].

Like Zimbardo’s approach to understanding PTSD as a disorder of time [36] some view anniversary reactions as a pathological response to trauma and loss [48–52] while more recent approaches expand anniversary reactions to understand that the process of coping with trauma and loss is rife with temporal triggers–or event/time specific relationships [33]. To our knowledge, this is one of the first studies to formally explore the relationship between temporal triggers–like markers in time- with the time perspective. However, given the important role of subjective time in the experience of markers in time, it stands to reason that one’s time orientation would be related. While we offer preliminary evidence of this association in our study, we note the need for additional research to explore this relationship more deeply.

### Limitations

There are three important limitations to consider when interpreting the results of our analysis. Firstly, our small sample size posed limitations regarding statistical power in our regression analysis. As a result, we were limited by the number of predictors and controls we could include in our models and note that more complex models may better explain outcomes associated with time perspectives, specifically including demographic covariates in regression models. However, we also note that being underpowered typically results in type II errors; therefore, identifying significant relationship in our small sample suggest that further exploration is warranted. Secondly, we acknowledge that our operationalization of PTG relies on a single construct conceptualization and does not take into consideration earlier noted criticisms of this approach. However, we note that using a total score of the PTGI is a standard practice in the literature and thus our analysis is consistent with traditional usages of the PTGI. Finally, the survey was publicly available, and some individuals responded more than once to the link. Duplicates were deleted when repeated email addresses were present (n = 15) or when qualitative responses clearly indicated copy and paste (n = 11). While every attempt to remove duplicate entries was made by the research team, we note the possibility that duplicate responses that were not clearly indicated (as noted above) may have been retained in the analysis.

### Implications and future directions

Despite these limitations, our results offer important preliminary insights into the utility of PTSD, PTG, and the endorsement of markers in time in influencing survivor’s time perspective. We encourage additional research with an emphasis on identifying the direction of these relationships (e.g. bi-directional) and the identification of important covariates (demographics, trauma/loss characteristics, other psychosocial variables) and possible mediators (e.g. social support) that may highlight the pathway to reducing PTSD, promoting PTG, and creating greater flexibility in navigating time perspectives.

In doing so, these lines of scholarship may enhance mental health practice with trauma affected and bereaved populations. More specifically, understanding the relationships among these constructs may help clinicians understand symptom clusters, and may also aid in targeted and individualized intervention approaches that uniquely address the context and experience of the survivor. Lastly, understanding the interaction of the individual with time is critical to develop a language for the survivor to articulate their experience of coping with trauma and loss. Recognizing that healing is inextricably linked with our perception of time is an important first step in more accurately reflecting the lived experiences of survivors in our scholarship and in our treatment approaches.
